# Improving Diagnostic Accuracy for Peripheral Neuropathy: Use of Tibial Nerve Somatosensory Evoked Potentials

**DOI:** 10.3390/jfmk11020208

**Published:** 2026-05-26

**Authors:** Miki Oka, Shozo Tobimatsu, Akira Yokote, Ayako Sakoda, Saeko Inamizu, Yuri Nakamura, Keiko Haro, Yuki Yanagihara, Yasutaka Iwanaga, Ken-Ichiro Yamashita, Jun-Ichi Kira

**Affiliations:** 1 Clinical Laboratory, Fukuoka Central Hospital, Fukuoka 810-0022, Japan; dt.0511and0908@hotmail.co.jp; 2 Faculty of Medicine, Fukuoka International University of Health and Welfare, Fukuoka 814-0001, Japan; 3 Department of Neurology, Brain and Nerve Center, Fukuoka Central Hospital, Fukuoka 810-0022, Japan; yokote_914@ihwg.jp (A.Y.); ayakosakoda@ihwg.jp (A.S.); nakayuri@ihwg.jp (Y.N.); harok@ihwg.jp (K.H.); tyukin@ihwg.jp (Y.Y.); iwanaga_y@ihwg.jp (Y.I.); kyamashita@ihwg.jp (K.-I.Y.); junkira@ihwg.jp (J.-I.K.); 4 Department of Neurology, Minohara Hospital, Sasaguri 811-2402, Japan; isaeko31@gmail.com; 5 Translational Neuroscience Research Center, Graduate School of Medicine, International University of Health and Welfare, Okawa 831-8501, Japan

**Keywords:** neuropathy, radiculopathy, sensory nerve function, electrophysiological testing

## Abstract

**Background**: We investigated whether combining a sural nerve sensory conduction study (s-SCS) and tibial nerve SEPs (t-SEPs) improves diagnostic accuracy for peripheral sensory neuropathy. **Methods**: We retrospectively reviewed 74 consecutive cases (114 lower limbs) of patients suspected of having neuropathy or radiculopathy who underwent s-SCSs and t-SEPs between July 2021 and December 2024. Abnormal S-SCSs were defined as a reduction in amplitude or a slowing of conduction velocity. Abnormal t-SEPs were defined as failure to evoke N20 or P37, or prolonged latency of either. **Results**: No cases showed s-SCS abnormalities with normal t-SEPs. Then, we classified the groups based on the combination of abnormality of s-SCSs and t-SEPs. Group 1 (G1) had normal s-SCSs and normal t-SEPs, which were observed in 31 limbs (27.2%). Group 2 (G2) had normal s-SCSs and abnormal t-SEPs, which were found in 45 limbs (39.5%). Subgroups of G2 included normal N20 with abnormal P37, abnormal N20 with normal P37, and N20/P37 abnormalities. Group 3 (G3) had abnormal s-SCSs with abnormal t-SEPs, which were seen in 38 limbs (33.3%). **Conclusions**: Electrophysiological testing reveals normal distal and proximal sensory nerves in G1, suggesting preserved sensory nerve function. The distal sensory nerves are normal in G2. However, abnormal N20/P37 and abnormal N20 with normal P37 indicate proximal sensory nerve involvement. Normal N20 with abnormal P37 indicates posterior column dysfunction. In G3, both the distal and proximal sensory nerve segments are abnormal. Therefore, adding t-SEPs to s-SCSs allows us to evaluate the full length of the peripheral nerves, which is useful for diagnosis and assessing treatment efficacy.

## 1. Introduction

Traditionally, the diagnosis of peripheral nerve disorders has depended on medical history and clinical examination [1,2,3]. Electrodiagnostic studies provide a quantitative assessment of disease severity and reveal the pathophysiology. They may also confirm the nerve injury’s location [4,5,6], which best categorizes peripheral neuropathy. One of the most common localizations is a diffuse, length-dependent process called distal symmetric polyneuropathy (DSP). Another common site of nerve injury is mononeuropathy, as in the case of carpal tunnel syndrome. Rare peripheral neuropathy locations include diffuse, non-length-dependent neuropathies and multiple mononeuropathies, such as chronic inflammatory demyelinating polyradiculoneuropathy (CIDP), vasculitic neuropathy, and diabetic neuropathy [2,3]. The main purpose of electrodiagnostic studies in polyneuropathy is to differentiate between axonal degeneration and demyelination, as clinical information alone cannot distinguish between these two processes [7]. Demyelination is generally indicated by decreased nerve conduction velocity (NCV), conduction block, or increased temporal dispersion [7]. Axonal loss is indicated by a reduced amplitude or area of the sensory nerve action potential (SNAP) or compound muscle action potential (CMAP). However, differentiating between demyelinating and axonal pathophysiologies using nerve conduction studies can sometimes be difficult [7].

CIDP is the most common form of autoimmune polyneuropathy. CIDP is characterized by the selective involvement of peripheral nerves, plexuses, or nerve roots [5,6]. In our neurology department, we routinely perform tibial nerve somatosensory evoked potential (t-SEP) testing on patients with polyneuropathy (DSP, CIDP, etc.) and/or radiculopathy to evaluate proximal sensory nerve function, the spinal entry zone, and the posterior column. We coincidentally noticed disagreement between the results of the sensory conduction study (SCS) of the sural nerve (s-SCS) and the t-SEP results: some patients showed normal s-SCSs with abnormal t-SEPs. We assumed that this was because s-SCSs cannot evaluate proximal sensory nerve damage, whereas t-SEPs are useful for assessing the function of the proximal sensory nerve and the spinal entry zone [8,9]. Although expert recommendations for the clinical use of SEPs did not specifically address peripheral neuropathies [10], the value of SEPs in neuropathy has been demonstrated by several studies published in the literature [11,12,13,14,15,16,17,18,19], especially in CIDP [12,13,14,15,16,17,18]. Therefore, we investigated whether combining s-SCSs and t-SEPs improves diagnostic accuracy for peripheral sensory neuropathy using a retrospective cohort design.

## 2. Materials and Methods

### 2.1. Study Design and Setting

We performed a retrospective observational study at Fukuoka Central Hospital, a 140-bed hospital with a neurological center located in southern Japan. The hospital performs approximately 250 motor and sensory nerve conduction studies and 250 evoked potential studies per year. We performed both s-SCSs and t-SEPs to evaluate various neurological disorders, such as CIDP and multiple sclerosis. Our institutional ethics committee approved this study (25-KH-045), waiving the requirement for individual patient consent due to its retrospective nature. Informed consent was obtained from all subjects involved in the study.

### 2.2. Patient Population

We conducted a retrospective review of 74 consecutive cases (24 females) of suspected peripheral neuropathy or radiculopathy. Their ages ranged from 27 to 91 years old. Table 1 shows a spectrum of peripheral nerve diseases with various etiologies. The most common causes are immune-mediated conditions (CIDP and Guillain–Barré syndrome (GBS)), as well as collagen vascular disorders associated with vasculitic neuropathy and radiculopathy. These patients underwent s-SCSs and t-SEPs at our institution between July 2021 and December 2024. We conducted a thorough evaluation of all patients prior to treatment. We excluded patients with myelitis or myelopathy who exhibited central nervous system dysfunction. We also excluded patients with neuropathy for whom T-SEPs were not performed.

### 2.3. Sural Nerve Conduction Study

A board-certified clinical neurophysiologist (SI) from the Japanese Society of Clinical Neurophysiology carried out s-SCSs with the assistance of a laboratory technician (MO) and three other doctors (AS, YN and SI; SI is a board-certified clinical neurophysiologist from the Japanese Society of Clinical Neurophysiology). s-SCSs were performed using a Neuropack X1 electromyograph system (Nihon Koden, Tokyo, Japan) with standard techniques. The skin temperature over the lateral malleolus was kept above 31 °C. The sural nerves were stimulated antidromically using a surface stimulator. The surface recording electrode was placed posterior to the lateral malleolus, and the reference electrode was placed 3 cm distally. The stimulation site was the posterior aspect of the calf, with the cathode placed 14 cm proximal to the recording electrode. A ground electrode was placed between the recording and stimulating electrodes. The stimulus intensity gradually increased until a supramaximal sensory response was obtained. Standard filter settings were used to record SNAPs, and responses were averaged at least ten times, or more, if necessary, until a stable waveform was obtained. Latencies were calculated from stimulus onset to the initial negative deflection for biphasic SNAPs or to the first positive peak for triphasic SNAPs to determine CV. Amplitudes were measured from the lowest positive peak to the negative peak. According to a previous publication, SNAP amplitudes of less than 3.8 µV and conduction velocities of less than 39.3 m/s were considered abnormal [20].

### 2.4. Somatosensory Evoked Potential Recording

A board-certified clinical neurophysiologist (ST) from the Japanese Society of Clinical Neurophysiology performed t-SEPs with the help of MO and three other doctors (AS, AY, and KH). SEPs were obtained by stimulating the posterior tibial nerve at the ankle at a frequency of 2 Hz [13,21,22]. The recording electrodes were placed over the 12th thoracic vertebra and Cz, which was 2 cm posterior to Cz according to the international 10–20 system. An electrode placed over the contralateral iliac crest and Fz was used as the reference. A Neuropack X1 amplifier (Nihon Kohden, Tokyo, Japan) with a bandpass of 5–2000 Hz was used, and 350 responses were averaged. At least two trials were superimposed to establish reproducibility. The peak latencies of the responses were measured: N20 (Th12) and P37 (sensory cortex) for tibial nerve SEPs. The central sensory conduction time (CSCT) was calculated as P37–N20. The normal mean values were 20.02 ms for N20, 36.91 ms for P37, and 16.88 ms for N20–P37 (CSCT), respectively [13,21,22]. Latencies exceeding the mean + 3 SD from the established normal values for SEPs were considered abnormal.

### 2.5. Criteria for Abnormal SCSs and SEPs

Abnormal s-SCSs were defined as an amplitude of less than 3.8 μV or a conduction velocity of less than 39.3 m/s [20]. An abnormal t-SEP was defined as a failure to evoke an N20 response, an N20 latency greater than 24.37 ms, a failure to evoke a P37 response, a P37 latency greater than 44.35 ms, or a CSCT greater than 21.83 ms [12,20].

### 2.6. Clinical Data Collection

Clinical information was extracted from electronic medical records. Board-certified neurologists from the Japanese Society of Neurology (AY, AS, YY, KH, YI, YN, KY, and JK) made diagnoses according to the Society’s established diagnostic criteria [1,2,3,4].

## 3. Results

**Figure 1:**
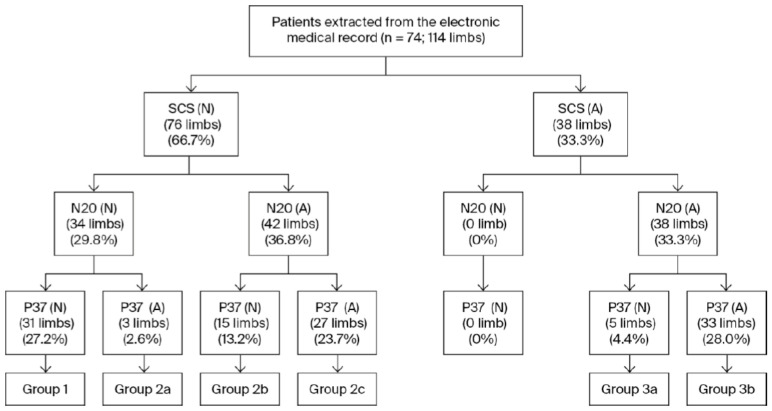
Flowchart of patients. Patients were categorized into three groups based on the results of s-SCSs and t-SEPs (N20/P37). Abbreviations: s-SCS, sural nerve sensory conduction study; t-SEPs, tibial nerve SEPs; N, normal; A, abnormal.

**Figure 2:**
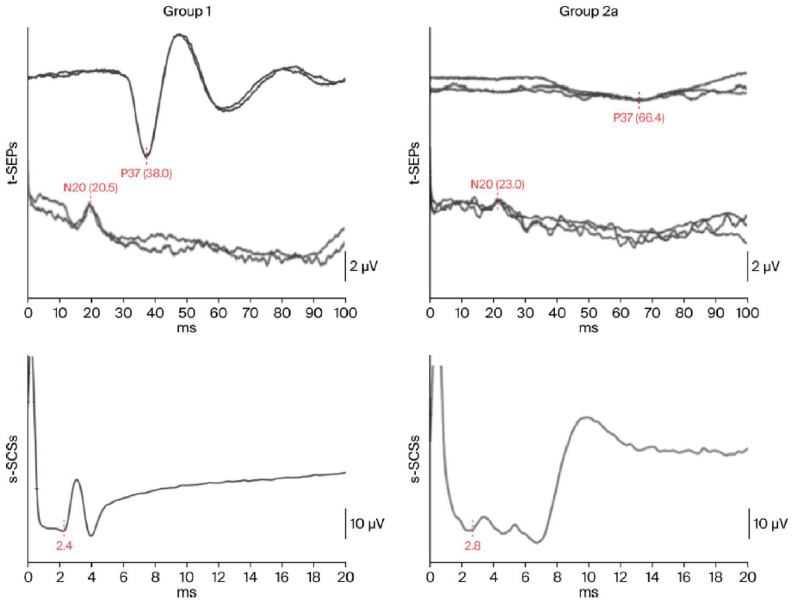
Illustrated cases of Groups 1 and 2a. A 50-year-old female patient with small-fiber sensory neuropathy showed normal s-SCSs and t-SEPs (Group 1). This suggests the relative preservation of large sensory fibers. A 39-year-old male patient with CIDP had normal s-SCSs and N20 but his P37 was prolonged and temporally dispersed (Group 2a). This indicates the dorsal column dysfunction.

**Figure 3:**
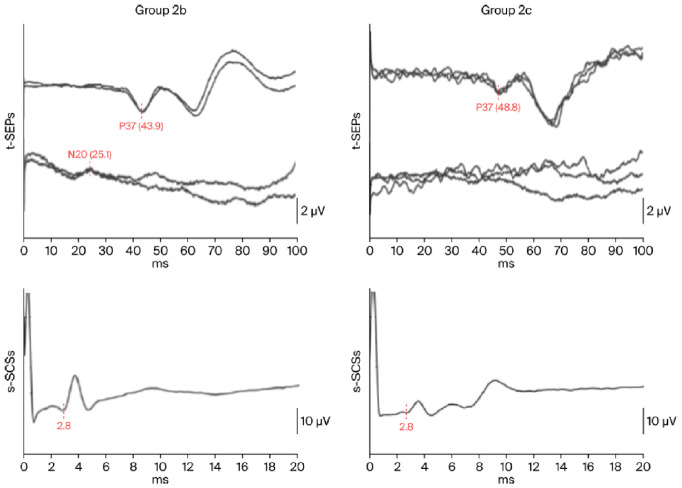
Illustrated cases of Groups 2b and 2c. A 66-year-old male patient with diabetic polyneuropathy had normal s-SCSs and prolonged N20 with normal P37 (Group 2b). A 77-year-old male patient with vitamin deficiency polyneuropathy had normal s-SCSs, as well as prolonged N20 and P37 (Group 2c). Groups 2b and 2c both suggest proximal sensory nerve damage.

**Figure 4:**
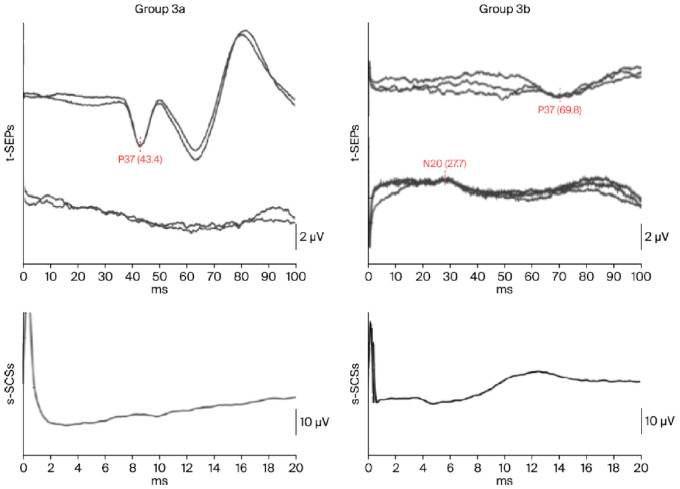
Illustrated cases of Groups 3a and 3b. A 75-year-old female patient with vasculitic neuropathy showed absent SNAP and prolonged N20 with normal P37 (Group 3a). A 65-year-old male patient with alcoholic neuropathy had absent SNAP and prolonged N20 and P37 responses (Group 3b). These findings suggest the presence of severe neuropathy caused by axonal dysfunction in the peripheral nerves.

Although we tested t-SEPs bilaterally in all 74 subjects, s-SCSs were not always examined bilaterally. Thus, the total number of s-SCSs examined was 114 limbs. Figure 1 summarizes the results of this study. Since 80 limbs showed abnormal N20 responses, CSCT was not included as an index of t-SEP abnormality. As expected, no cases showed s-SCS abnormalities with normal t-SEPs (Figure 1). From Figure 1, three groups were found based on the results of s-SCSs and t-SEPs. Group 1 had normal s-SCSs and normal t-SEPs, which were observed in 31 limbs (27.2%) (Figure 2). Group 2 had normal s-SCSs with abnormal t-SEPs, which were observed in 45 limbs (39.5%). Within Group 2, there were three subgroups: normal N20 with abnormal P37 (Group 2a, 2.6%; Figure 2); abnormal N20 with normal P37 (Group 2b, 13.2%; Figure 3); and abnormal N20 with abnormal P37 (Group 2c, 23.7%; Figure 3). Group 3 had abnormal s-SCSs with abnormal t-SEPs. This combination was observed in 38 limbs (33.3%; see Figure 4): five limbs showed normal P37 (Group 3a), while 33 limbs had abnormal P37 (Group 3b). Abnormal N20 with normal P37 was found in 20 limbs (17.5%) in combination with Groups 2b and 3a.

## 4. Discussion

The diagnostic utility of t-SEPs in demyelinating diseases, such as multiple sclerosis, is well-established. In particular, prolonged CSCT indicates abnormal t-SEPs [21,23]. This study examined the usefulness of t-SEPs for diagnosing and classifying peripheral neuropathies and radiculopathies. However, CSCT was not included as an index of abnormality because 80 limbs showed abnormal N20 responses. This finding restricted the use of the CSCT parameter for subgrouping patients. The utility of t-SEPs was best demonstrated in patients with abnormal t-SEPs with normal s-SCSs (39.5%, Group 2), for whom the presence of proximal sensory nerve damage could not be confirmed by conventional nerve conduction studies. Group 2 appears to be divided into three subtypes: normal N20 with abnormal P37 (Group 2a, Figure 2 left); abnormal N20 with normal P37 (Group 2b, Figure 3 left); and abnormal N20 and P37 (Group 2c, Figure 3 right). Group 2a indicates the dorsal column dysfunction, while Groups 2b and 2c suggest proximal sensory nerve damage. However, it is unclear why t-SEPs can be recorded over the scalp when peripheral responses are abnormal in Groups 2b and 3a (17.5%). Presumably, there is some reorganization and amplification of impulse traffic within the central nervous system [8,9,24]. Peripheral SNAPs may not be recorded when peripheral impulse traffic is dispersed; however, there may be central synchronization at different synaptic levels to amplify the volleys. In accordance with the “cortical amplification” theory [8,9,24], we found that t-SEPs could be obtained even when the N20 is abnormal. An alternative interpretation is that it is phase cancellation and temporal dispersion of the N20 of t-SEPs but not the P37 [25,26]. Overall, our findings suggest that t-SEP studies may be an important ancillary neurophysiological tool for classifying sensory neuropathies and diagnosing non-length-dependent polyneuropathies, such as CIDP, alongside conventional NCS.

We found that of the 76 limbs with normal s-SCSs, normal t-SEPs were observed in 31 limbs (27.2%, Group 1, Figure 2 left). Upon reviewing the medical records, we discovered that the motor conduction velocity of the tibial nerve was abnormal in five limbs, indicating the presence of motor-dominant neuropathy. All four cases of GBS were included in this group. The remaining patients with normal s-SCSs and t-SEPs may not have large fiber neuropathy. Conversely, all patients with abnormal s-SCSs had abnormal t-SEPs (Group 3). This finding suggests the presence of severe, length-dependent neuropathy caused by severe axonal dysfunction of the peripheral nerve. Electrophysiological differentiation between demyelinating and axonal pathologies can be difficult using peripheral nerve conduction studies alone. Thus, the combined use of s-SCSs and t-SEPs enables differentiation between length-dependent and non-length-dependent neuropathies.

The value of SEPs in CIDP is well established [12,13,14,15,16,17,18]. In accordance with previous study results, nine out of ten CIDP patients and two patients with combined central and peripheral demyelination (CCPD) were classified as Groups 2 and 3. Interestingly, one CIDP patient (Group 1) showed abnormal N20 with normal P37 in left t-SEPs, though a left SCS was not performed. The 2010 European Federation of Neurological Societies (EFNS)/Peripheral Nerve Society (PNS) criteria [27] and the 2021 European Academy of Neurology (EAN)/PNS guidelines for diagnosing CIDP do not recommend using SEPs for electrodiagnosis [28]. CIDP is confirmed if there is at least one demyelinating feature in two motor nerves and is only “possible” if demyelinating features are identified in only one nerve. The EAN/PNS diagnostic criteria also require sensory nerve conduction study (NCS) abnormalities in two or more nerves, which are defined as prolonged distal latency, reduced SNAP amplitude, or slowed conduction velocity. Doneddu et al. [29] performed a comparison of the diagnostic accuracy of the 2010 EFNS/PNS and 2021 EAN/PNS criteria for CIDP. They found that the EAN/PNS criteria are more specific for probable or definite CIDP but less sensitive than the EFNS/PNS criteria. Thus, they concluded that more extensive nerve conduction studies improve the diagnostic sensitivity of the EAN/PNS criteria while maintaining a high specificity. Based on the results of previous studies [12,13,14,15,16,17,18] and our study, t-SEPs allow for a more thorough evaluation of the peripheral nervous system than conventional techniques.

The present study has several limitations. First, the wide variety of etiologies of neuropathy and radiculopathy is a significant limitation of the present study. This heterogeneity complicates interpretation of the electrophysiological findings. However, our study clearly demonstrated the effectiveness of using s-SCSs and t-SEPs together on the electrodiagnosis of neuropathy. Second, we used a narrative approach to describe the differences among the three groups. Including clinical-electrophysiological correlations would considerably improve the proposed classification’s clinical relevance. Finally, we did not perform the statistical analysis due to the small number of patients in each group. Therefore, a prospective cohort study with a large sample size focusing on length-dependent and non-length-dependent neuropathies is necessary.

## 5. Conclusions

This retrospective and exploratory study clearly demonstrates the clinical utility of combining s-SCSs and t-SEPs for evaluating the entire sensory pathway. Adding t-SEPs to s-SCSs allows for an electrophysiological evaluation of both the distal and proximal segments of peripheral nerves and is useful for diagnosing and assessing treatment efficacy. Therefore, t-SEPs appear to be a second-line test that provides valuable and complementary information on the central and proximal nervous systems.

## Figures and Tables

**Table 1:** Etiologies of the patients.

Immune-mediated neuropathy	n = 16
Vasculitic neuropathy	n = 15
Radiculoneuropathy	n = 12
Vitamin deficiency neuropathy	n = 4
Viral infectious neuropathy	n = 4
Diabetic neuropathy	n = 4
Toxic neuropathy	n = 3
Inherited neuropathy	n = 1
Unclassified	n = 15

## Data Availability

The data that support the findings of this study are available upon request from the corresponding author. The data are not publicly available due to privacy or ethical restrictions.

## Abbreviations

The following abbreviations are used in this manuscript:

CCPD	Central and peripheral demyelination
CIDP	Chronic inflammatory demyelinating polyradiculoneuropathy
CMAP	Compound motor action potential
CSCT	Central sensory conduction time
DSP	Distal symmetric polyneuropathy
EFNS	European Federation of Neurological Societies
EAN	European Academy of Neurology
G	Group
GBS	Guillain–Barré syndrome
NCS	Nerve conduction study
NCV	Nerve conduction velocity
PNS	Peripheral Nerve Society
SCS	Sensory conduction study
SEPs	Somatosensory evoked potentials
SNAP	Sensory nerve action potential
s-SCS	Sural nerve SCS
t-SEPs	Tibial nerve SEPs
